# Comparison of *in vitro* erosion protocols in bovine teeth to simulate natural erosion lesion: analysis of mechanical properties and surface gloss

**DOI:** 10.1590/1678-7757-2018-0107

**Published:** 2019-01-07

**Authors:** Mariana Dias Moda, Ticiane Cestari Fagundes, Eduardo Bresciani, André Luiz Fraga Briso, Paulo Henrique Dos Santos

**Affiliations:** 1 Univ. Estadual Paulista, Faculdade de Odontologia de Araçatuba, Departamento de Odontologia Restauradora, Araçatuba, São Paulo, Brasil; 2 Univ. Estadual Paulista, Instituto de Ciência e Tecnologia, Departamento de Odontologia Restauradora, São José dos Campos, São Paulo, Brasil; 3 Univ. Estadual Paulista, Faculdade de Odontologia de Araçatuba, Departamento de Materiais Odontológicos e Prótese, Araçatuba, São Paulo, Brasil

**Keywords:** Citric acid, Dentin, Tooth erosion, Pepsin A

## Abstract

**Objective:**

The aim of this study was to compare two *in vitro* erosion protocols, in which one simulates *in vivo* conditions experienced by patients with gastroesophageal disorders or bulimia (HCl-pepsin protocol), and the other simulates the diet of an individual who consumes a high volume of erosive beverages (citric acid protocol). In addition, the mechanical properties and surface gloss of eroded human dentin were compared with those of sound human dentin.

**Materials and Methods:**

Blocks of cervical dentin were used: sound human dentin (n=10), human dentin with erosive lesions (n=10), and bovine dentin (n=30). Twenty bovine blocks were subjected to either of two erosion protocols (n=10/protocol). In the first protocol, samples were demineralized using HCl-pepsin solution, then treated with trypsin solution. In the second protocol, samples were demineralized with 2% citric acid. Toothbrushing was performed in both protocols using a toothbrushing machine (15 s with a 150 g load). Ten bovine dentin blocks were not subjected to any erosive treatment. All samples of bovine and human dentin were analyzed to obtain Martens hardness values (MH), elastic modulus (Eit*) and surface gloss. One-way ANOVA and Tukey's test were performed to analyze the data (α=0.05).

**Results:**

Sound human and eroded human dentin groups showed similar MH and Eit* values (p>0.05); however, sound human dentin showed a higher surface gloss value when compared to eroded human dentin (p<0.05). Sound bovine dentin and HCl-pepsin-treated bovine dentin treatments resulted in similar values for both MH and Eit* (p>0.05), but HCl-pepsin-treated bovine dentin and citric acid-treated bovine dentin resulted in lower surface gloss than sound bovine dentin (p<0.05).

**Conclusions:**

The HCl-pepsin protocol modified bovine dentin properties that could be similar to those that occur on human dentin surfaces with erosive lesions.

## Introduction

The occurrence of non-carious cervical lesions (NCCL) is a very common clinical situation, resulting from multifactorial etiology.^1^ ^,^ ^2^ ^,^ ^3^ Establishing differentiation among NCCL types is difficult because most cases involve the association of abfraction, attrition, erosion, and abrasion.^4^ ^,^ ^5^ Acids from gastroesophageal disorders, foods and drinks play key roles in the development of erosive lesions, which may cause irreversible loss of dental tissue.^1^ ^,^ ^2^ ^,^ ^6^ ^,^ ^7^

Given this context, dentin is a complex tissue which undergoes constant change due to the network of tubules that extend from the pulp to the dentinoenamel junction; thus, being closely related to the pulp.^8^ ^,^ ^9^ Dentin hypersensitivity is common in cases of erosion.^1^ The erosion process starts in the peritubular dentin, which shows a greater degree of mineralization and involves dentinal tubules.^10^ Following, demineralization of hydroxyapatite crystals occurs in the intertubular dentin, thus exposing the collagen fibrils of the organic matrix.^10^ In addition, the erosive process can involve metalloproteinases, natural constituents of dentin that function in the degradation of collagen fibers, which may accentuate the erosion process by degrading the collagen matrix.^11^ Some modifications occur in eroded dentin areas, such as changes in mechanical properties^12^ and brightness.^13^

Due to the high incidence of *in vivo* dental erosion, there is a great need for protocols and studies that accurately reproduce the erosive process *in vitro*, to facilitate the better understanding of dentin tissue.^4^ ^,^ ^14^ However, simulating all complex conditions that occur in the oral environment is very difficult^4^. In addition, the use of enzymes or acids to cause *in vitro* erosion requires optimization to ensure that the outcomes have similar characteristics to *in vivo* lesions^4^. Some prior studies have used gastrointestinal tract enzymes to simulate gastroesophageal disorders.^6^ ^,^ ^7^ Another *in vitro* protocol involves the use of citric acid, a key ingredient in some beverages, which are associated with tooth erosion.^15^ ^,^ ^16^

However, the literature lacks *in vitro* protocols that accurately simulate clinical conditions of erosive tooth wear. Therefore, the objective of this study was to compare two *in vitro* erosion protocols, in which one simulates *in vivo* conditions experienced by bulimia patients (HCl-pepsin protocol), and the other simulates the diet of an individual who consumes high volumes of erosive beverages (citric acid protocol); these protocols reproduce alterations of human dentin subjected to natural erosive lesions. The second objective was to compare the mechanical properties and surface gloss of eroded human dentin and sound human dentin. Two null hypotheses were tested: (1) there would be no differences in mechanical properties and surface gloss between sound and eroded human dentin; (2) there would be no differences in mechanical properties and surface gloss of bovine dentin after exposure to two *in vitro* erosion protocols when compared to sound bovine dentin.

## Materials and methods

### Sample preparation

Twenty human pre-molars and lower incisors [sound human dentin (n=10) and human dentin with erosive lesions (n=10)] were collected from patients at a private clinic; the clinician who performed the surgical procedures provided information regarding the type of lesion in each case. Moreover, the clinician was able to detect these lesions through anamnesis, in which the patients reported their eating habits (ingestion of acidic beverages) or gastroesophageal disorders. All patients provided written informed consent to donate their teeth for the study. The research project was approved by the local Human Research Ethics Committee, #32545114100005420. Additionally, 30 bovine teeth were used in this study. All teeth were cleaned and stored in 0.1% thymol solution. The animal portion of this research project was approved by the local Animal Research Ethics Committee, #2016-00150.

Blocks of cervical dentin were obtained by using a slow-speed diamond saw (Isomet 2000, Buehler, Aurora, OH, USA). The blocks (4x4 mm) of sound human and bovine dentin were obtained by cutting into the dentin-enamel junction and extending up to 4 mm of the root dentin. Blocks from the erosive lesion group were restricted to the lesion area. All dentin blocks were mounted on acrylic bases and polished (Arotec APL 4, Cotia, SP, Brazil) with aluminum oxide abrasive papers (600-, 800- and 1200-grit). Final polishing was performed with felt discs embedded in a 1-μm diamond polishing suspension (Extec Corp., Enfield, CT, USA). Between each polish with abrasive paper, dentin blocks were ultrasonically cleaned (Cristófoli, Campo Mourão, PR, Brazil) in distilled water for 10 min. For eroded human dentin, after being mounted on the acrylic bases, only the edges were removed during the flattening process. The central features of these lesions remained unchanged, preserving the tissue characteristics for the analysis.

All blocks of human dentin (sound or eroded) and 10 blocks of bovine dentin were stored at 100% relative humidity for 2 weeks in an incubator (ECB-2. Adamo Products for Laboratory Ltda., Piracicaba, SP, Brazil) at 37°C. The other 20 bovine dentin blocks were treated with the erosion protocols, as described below. All samples had their Martens hardness (MH) values, elastic modulus (Eit*), and surface gloss determined.

### Erosion protocols

Twenty bovine blocks were treated with either of two erosion protocols (n=10/protocol):

HCl-pepsin protocol:^7^ 10 bovine specimens were cyclically demineralized over 9 days. HCl-pepsin solution was used to perform six demineralization cycles (2 min each) per day, under smooth stirring (30/min) in a water bath at 37°C. The demineralizing solution was prepared by dissolving 5 mg/ml NaCl in distilled water and adjusting the pH to 1.6 with HCl. Finally, 1.5 mg/ml pepsin (4800 U/ml; P-6887, pepsin from porcine gastric mucosa, Sigma-Aldrich, Seelze, Germany) was added to the HCl-pepsin solution. After each erosive process, all specimens were treated with a trypsin solution that was prepared by dissolving 2000 BAEE units/ml trypsin (T-9201, trypsin from bovine pancreas, Sigma-Aldrich, Seelze, Germany) in a mineral salt solution for 10 min. The trypsin solution contained 4.08 mM H_3_PO_4_, 20.10 mM KCl, 11.90 mM Na_2_CO_3_, and 1.98 mM CaCl_2_; maintaining a 6.7 pH. This solution was also used for sample storage (up to 18 h overnight) and for the composition of the slurry. After the first and last trypsin treatments, specimens were mechanically brushed for 15 s with a fluoride-free toothpaste (slurry with remineralization solution, 1:3 w/w). A brushing machine (150 g axial load, five strokes/s; Elquip, São Carlos, SP, Brazil) was used for this process. After each intervention, specimens were thoroughly rinsed for 1 min with distilled water.

Citric acid protocol:^15^ 10 bovine dentin blocks were immersed in 2% citric acid (pH 2.8) for 5 min, under smooth stirring (30/min) in a water bath at 37°C, over 5 days. After each erosion cycle, abrasion was performed on the blocks by using a mechanical brushing machine (150 g axial load, five strokes/s; Elquip, São Carlos, SP, Brazil). Brushing was performed for 15 s each time, by using a dentifrice slurry (diluted 1:3 w/w in distilled water) containing carboxymethylcellulose, sodium saccharin, glycerol, peppermint oil, and water, in which fluoride was incorporated. The fluoride concentration was 4500 μgF/g. This process was repeated every 2 h, for a total of four cycles each day.

**Figure 1:**
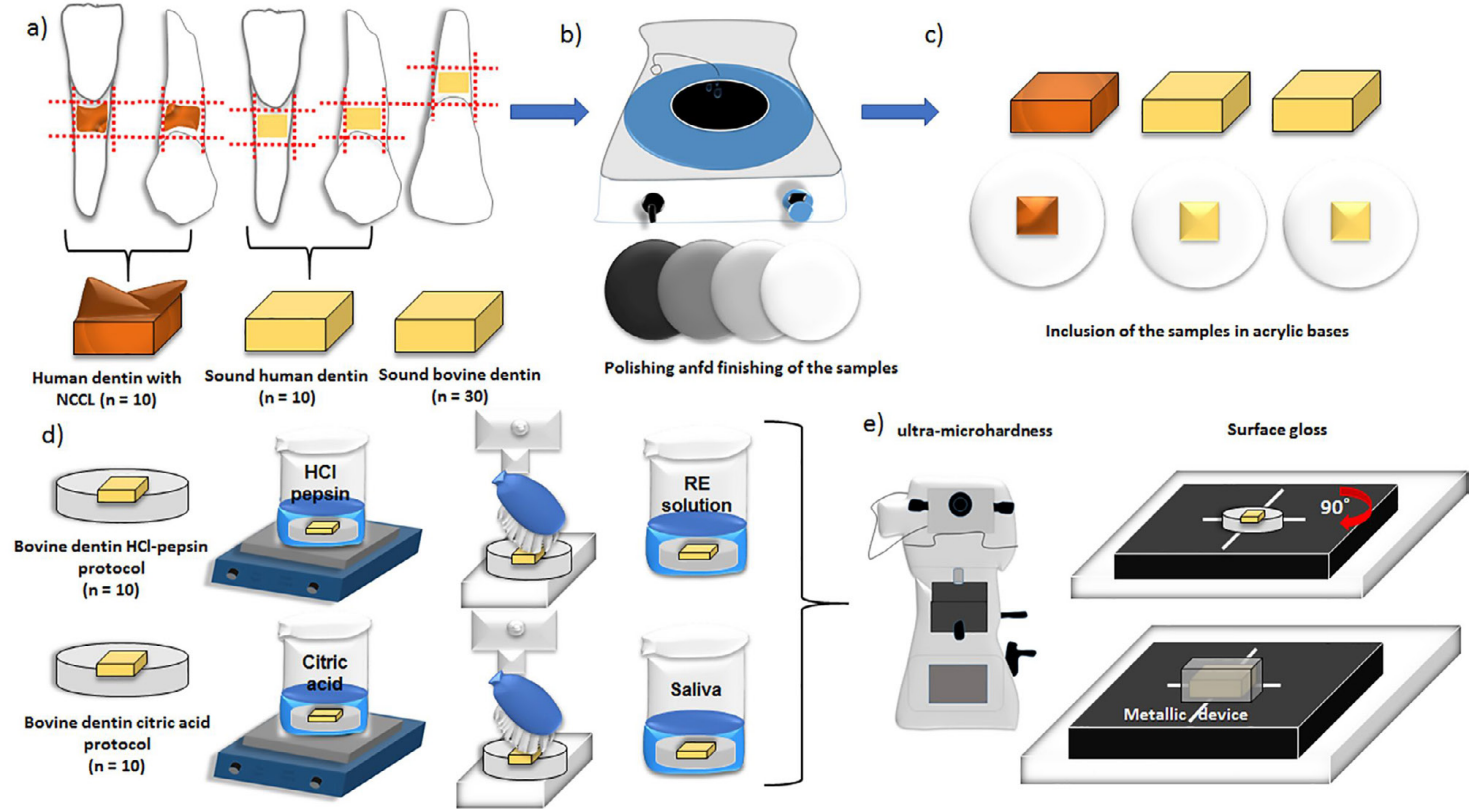
(a) Blocks of root dentin; n=10 human dentin with NCCL (incisors and premolars); n=10 sound human dentin (incisors and premolars); n=30 sound bovine dentin (4x4 mm); b) Polishing of samples in automatic polishing machine with # 600, # 800, # 1200 and 1-μm diamond polishing suspension; c) Inclusion of all samples in acrylic resin bases; d) Twenty bovine dentin blocks were subjected to either of two erosion protocols. HCl-pepsin protocol (n=10) and citric acid protocol (n=10); e) Analysis of the mechanical properties (MH and Eit*) and surface gloss of all samples. NCCL=non-carious cervical lesions

All samples had their mechanical properties and surface gloss evaluated. The complete experimental design is illustrated in Figure 1. Sample size was based on previous studies for both mechanical properties^17^ and gloss surface^18^ analysis.

### Evaluation of mechanical properties

For each sample, MH and Eit* measurements were performed using a digital dynamic ultra-microhardness tester (DUH-211S, Shimadzu, Kyoto, Japan) with a Vickers indenter tip under a 500 mN load, at 70.0670 mN/s loading speed for 5 s of holding time. Five indentations were made in the central region of each sample with 100 μm between each one. Mean MH and Eit* measurements were obtained for each sample.

The MH value (N/mm^2^) is defined as the maximum force (F max) divided by the surface area of the indenter, multiplied by the squared penetration depth (h):

$$\text{MH}=\frac{\text{F}\max}{26.43\;{\text{h}}^{2}}$$

The Eit* value was calculated according to the following equation:

$$\frac{1}{\text{Er}}=\frac{\left(1-v^{2}\right)}{Eit*}+\frac{\left(1-vi^{2}\right)}{Ei}$$

Here *v* and *vi* are Poisson's coefficient (defined as the property between the specific transverse and longitudinal deformations) of the sample and indenter, respectively, and *Ei* is the elastic modulus of the indenter.

### Surface gloss

Surface gloss analysis was performed by using the Micro-Gloss 60 device, Novo-Curve Glossmeter (Novo-Curve, Rhopoint TM, England), on a 2x2 mm area with 60° geometry (light incidence). Surface gloss measures were expressed in gloss units (GU) and ranged from 0 to 100. The measuring principle of this device is based on light beam incidence at 60° to the object surface, where the glossmeter measures the intensity of the reflected light at 60° and compares it to a reference value (polished black standard −1.567 refractive index). The equipment was calibrated prior to each analysis using the reference standard. Three readings were made at the center of each specimen, which was then turned 90°, calculating a mean to obtain a single value for each specimen. The positioning of specimens at the center of the device was guided by the intersection of the white lines marked on its sole plate. For each reading, specimens were covered by a metallic device to avoid interference from external light.

Specimens were analyzed in dry conditions because of the formation of water film, to avoid changes in the refractive index of the samples.

### Statistical analysis

All statistical analyses were performed with the StatView statistical software version 5.0.1 (SAS Institute, Cary, NC, USA). Normal distribution of data was confirmed with the Shapiro-Wilk test. Homogeneity of variances was checked by using Bartlett's test. One-way ANOVA was used for data analysis and Tukey's test was used for multiple comparisons (α=0.05). The power analysis was done for all non-significant results using the OpenEpi webpage.

## Results

Sound and eroded human dentin showed similar MH and Eit* values (Table 1), with no statistically significant differences (p>0.05). The power analysis was 100% for MH and 29% for Eit*. However, surface gloss showed higher values for sound human dentin than for eroded human dentin (p<0.05). Table 1 summarizes all data regarding mechanical properties and surface gloss.

**Table 1:** Means and standard deviations of MH (GPa), Eit* (GPa) and surface gloss (GU) of sound and NCCL human dentin

Groups	MH	Eit*	Surface gloss
Human dentin with NCCL	1.5±0.09^A^	38.7±2.2^A^	54.4±2.9^B^
Sound human dentin	1.7±0.07^A^	37.9±1.3^A^	65.7±2.5^A^

* Different letters in columns indicate that values are significantly different from each other (<0.05). NCCL=non-carious cervical lesions

Sound bovine dentin was not statistically different from bovine dentin that were subjected to the HCl-pepsin protocol considering MH and Eit* values (p>0.05 for both). Bovine dentin that underwent the citric acid protocol showed the lowest MH and Eit* values when compared to both sound and HCl-pepsin-eroded bovine dentin (p<0.05). These values are summarized in Table 2. Sound bovine dentin showed higher surface gloss values than other groups (p<0.05, Table 2). Regarding surface gloss, no statistical differences were found between bovine dentin that were subjected to either of the erosion protocols (p>0.05).

**Table 2:** Means and standard deviations of MH (GPa), Eit* (GPa) and surface gloss (GU) of sound and eroded bovine dentin

Groups	MH	Eit*	Surface gloss
Bovine dentin with citric acid protocol	0.7±0.04^B^	20.5±1.2^B^	60.7±2.8^B^
Bovine dentin with HCl-pepsin protocol	1.1±0.04^A^	28.3±0.9^A^	60.3±1.7^B^
Sound bovine dentin	1.2±0.05^A^	29.2±1.0^A^	76.8±2.3^A^

* Different letters in columns indicate that values are significantly different from each other (p<0.05)

## Discussion

According to the results obtained in this study, MH and Eit* values for both sound and eroded human dentin showed no statistical differences (Table 1). This may be a result of reparative or sclerotic processes that occur in eroded human dentin.^10^ ^,^ ^19^ After experiencing erosion, odontoblasts within dentinal tubules can start the formation of tubular dentin with a new morphology,^10^ ^,^ ^20^ create minerals that occlude the dentinal tubules^10^ ^,^ ^19^ and form atubular dentin.^10^ These processes may contribute to the maintenance of the mechanical properties of eroded human dentin, in relation to sound human dentin. The demineralized surface layer was softened after the erosive challenge^21^ and could be removed by mechanical abrasions, such as toothbrushing,^21^ ^,^ ^22^ increasing the hardness values of eroded human dentin.

In general, sound human dentin showed higher MH and Eit* values than sound bovine dentin. This result is consistent with a previous study^23^ that found greater hardness values in human dentin because bovine dentin has a higher density of dentinal tubules.^23^ ^,^ ^24^ Despite this finding, some authors consider bovine dentin to be suitable for *in vitro* experiments, especially when combined erosion and abrasion are applied.^25^ Furthermore, some studies found no significant differences between human dentin and demineralized and mineralized bovine dentin in conventional hardness tests.^26^

When comparing the citric acid and HCl-pepsin protocols, the citric acid protocol was found to be more aggressive in terms of dentin mineral dissolution (Table 2). Citric acid chelates with calcium ions and may dissolve the smear layer of dentinal tubules,^15^ thus contributing to the reductions in mechanical properties found in this study (Table 2). Moreover, the exposure of a demineralized organic surface layer^27^ could also result in lower mechanical properties, especially with low loads. According to Wiegand, et al.^4^ (2011), there are considerable differences among the various studies that use erosion protocols, such as pH values, durations of erosive-abrasive cycles, toothpaste composition, and abrasive cycles.^4^ Other factors may contribute to mild or severe erosion, including the degree of stirring of the samples in erosive solutions, as well as the presence or absence of a smear layer.^4^ ^,^ ^14^ The protocols used in this study were chosen because the first simulated patients with gastroesophageal disorders or bulimia, and the second reproduced the diet of an individual who consumes high volumes of erosive beverages.

Pepsin can degrade the organic matrix by up to 25% without influencing mineral loss.^6^ Moreover, the remineralizing agent, consisting of a trypsin solution (also used for sample storage and slurry formation), is supersaturated when considering hydroxyapatite.^7^ These facts may explain the modified reduction in mechanical properties of specimens that were subjected to the HCl-pepsin protocol when compared to the citric acid protocol, and may also explain the lack of difference from sound bovine dentin (Table 2). Another important factor is that proteoglycans (PG) and glycosaminoglycan (GAG) form supramolecular aggregates that are interconnected with the collagen network; these can regulate the behavior of the extracellular matrix.^28^ Proteoglycans may act in the regulation of osmotic and hydrostatic pressure, as well as in the elastic behavior of the adjacent dentinal tubules.^28^ Similarly, PGs and GAGs bind primarily to the extracellular matrix and can regulate extracellular fluid.^28^ The dentino-cemental complex were degraded by digestive enzymes to investigate the influence of PGs and GAGs. Trypsin was used to remove PGs, and their absence decreased the resilience of the dentin by approximately 75% under stress conditions.^28^ The absence of PGs decreased the strength and ductility of the extracellular matrix, while the absence of GAGs decreased hardness and the elastic modulus.^28^ However, in our study, a minimal decrease in mechanical properties was observed for dentins that were subjected to the HCl-pepsin protocol, with no difference from sound bovine teeth (Table 2). In addition, the abrasive load used for brushing (150 g) may not be able to remove the demineralized organic matrix. A study of different abrasive loads found that a toothbrushing abrasion load of up to 4 N (approximately 400 g) was unable to remove the remaining organic matrix and protect the underlying dentin; therefore, it could not decrease the progression of the erosion process.^29^

*In vitro* protocols do not expose the samples to salivary dynamics that occur in the oral cavity;^4^ importantly, oral buffering dynamics that could minimize the mineral loss process.^10^ ^,^ ^23^ Although saliva contains enzymes that can degrade the organic matrix and accentuate the erosion process, it contains neutralization mechanisms and promotes remineralization of the substrate.^11^ ^,^ ^30^ Furthermore, *in vivo* erosion, which is strongly linked to the individual's lifestyle, is not continuous and standardized, as occurs in *in vitro* protocols.^10^ ^,^ ^23^ To simulate erosive lesions more accurately the erosion should be similar to the native pattern found in human dentin. The bovine dentin that was subjected to the HCl-pepsin protocol was exposed to enzymes that are natively in contact with the oral cavity when vomiting, or even in cases of reflux. This erosion protocol resulted in the modification of dentin properties that could be similar to those that occur in eroded human surfaces.

The methods used in this study aimed to quantify the mechanical behavior of dentin in different situations. Using conventional micro-hardness tests would not have been adequate, since the substrate presents elasticity due to possible contraction of the exposed organic matrix, and other conditions could alter the micro-hardness values.^31^ Indentations made with very low loads are the most appropriate method to evaluate dentin.^30^ When lower loads are applied under the indenter tip, dentin surfaces can be measured more adequately during early stages of erosion.^31^ Ultra-microindentation with the Vickers diamond was used in this study. A low load (500 mN) was used to allow greater sensitivity to small variations of the eroded substrate. Another advantage of the ultra-microhardness test is that mechanical properties can be evaluated considering both elastic and plastic deformations.^17^

Eroded human dentin showed lower surface gloss than sound human dentin (Table 1), rejecting the first null hypothesis of this study. Sound bovine and human dentin groups were polished and not subjected to any erosive protocol; therefore, their surface gloss values represent the initial unaltered values. Both erosive protocols decreased the surface gloss of bovine dentin when compared to sound bovine teeth (Table 2). Thus rejecting the second null hypothesis of this study. Ganss, et al.^10^ (2014) showed that after an *in vitro* erosion protocol the specimens were softened and dull. This corroborates the results obtained in this study, which showed loss of surface gloss regardless of the erosive protocol employed. Both *in vitro* erosive protocols resulted in similar decreases in surface gloss values. Nonetheless, when considering the mechanical properties, the modifications in dentin caused by the HCl-pepsin protocol were closer to modifications that occur in natural lesions than changes caused by the citric acid protocol. Namely, the comparison of the mechanical properties (MH and Eit*) of sound human dentin and eroded human dentin showed that they were similar (Table 1), as is the case for sound and HCl-pepsin-treated bovine dentin (Table 2). Other studies, such as profilometry and scanning electron microscopy, should be performed to confirm these results.

Although baseline values are important to assure that all samples were similar to mineral content, the lack of baseline and ΔMH measurements can be considered a limitation of this study, since it was not possible to conduct baseline measurements in the human dentin with NCCL before the lesions had occurred.

## Conclusion

Bovine dentin that were subjected to the HCl-pepsin protocol, which uses enzymes present in the gastrointestinal tract, showed similar mechanical properties (MH and Eit*) when compared to sound bovine teeth and a decrease in surface gloss values. Similarly, eroded human dentin showed similar mechanical properties and less surface gloss when compared to sound human dentin.
